# PET/CT used in the evaluation of pulmonary nodules suspicious for lung cancer in regions where infectious lung disease is endemic: to be or not to be?

**DOI:** 10.1590/0100-3984.2016.49.3.ce1

**Published:** 2016

**Authors:** Bruno Hochhegger

**Affiliations:** 1 Universidade Federal de Ciências da Saúde de Porto Alegre (UFCSPA), Porto Alegre, RS, Brazil.

*Dear Editor*,

The assessment of patients with suspected lung malignances^(1-4)^ has routinely included morphological imaging evaluation, with either chest X-rays or chest computed tomography (CT). In addition-although not diagnostic in character-^18^F-fluorodeoxyglucose positron emission tomography (FDG-PET), bone scintigraphy, and (occasionally) somatostatin receptor scintigraphy have been increasingly incorporated into daily practice in recent decades, providing physicians with useful and complementary information on the functional characteristics of lesions^(5,6)^. More recently, the emergence of combined PET/CT imaging has greatly aided the investigation of lung cancer by allowing even better delineation of areas with increased tracer uptake. This modality has helped radiologists avoid the technical difficulties that arose from the independent combination of PET and CT examinations, which resulted in substantial artifacts.

Many patients with early stage lung cancer will present with a solitary pulmonary nodule (SPN), defined as a single spherical or oval lesion that is less than 3 cm in diameter and is completely surrounded by pulmonary parenchyma without accompanying atelectasis or lymph node enlargement^(5,6)^. A very important step in investigating the etiology of an SPN is to determine whether it is benign or malignant in nature. In addition, PET/CT has been shown to be an accurate tool for the work-up of SPNs and for lung cancer staging, by improving the detection of metastatic disease, guiding therapy, and allowing clinical outcomes to be predicted^(5-7)^. However, there are a number of pitfalls to be considered during the assessment of SPNs with PET. In patients with inflammatory conditions or infections-such as bacterial or fungal infections; granulomatous diseases (tuberculosis, sarcoidosis, histoplasmosis, etc.); and pyogenic abscesses-there is a greater likelihood of higher metabolic activity due to increased granulocyte or macrophage activity, and such comorbidities have become a cause for great concern in some regions of Brazil^(8-10)^.

In a recent study published in **Radiologia Brasileira**, Mosmann et al.^(11)^ reviewed the evaluation of SPNs, in order to discuss the current role of FDG-PET (addressing its accuracy and cost-effectiveness) and to detail the current recommendations for the examination in this scenario. However, the authors did not focus on the applicability of FDG-PET in areas endemic for infectious granulomatous diseases. Deppen et al.^(12)^ performed the most recent and biggest meta-analysis about the diagnostic accuracy of FDG-PET for pulmonary nodules suspicious for lung cancer, comparing the accuracy of the test in regions where infectious lung disease is endemic with that reported for regions where such disease is rare^(8)^. The pooled (unadjusted) sensitivity and specificity were 89% (95% CI: 86-91%) and 75% (95% CI: 71-79%), respectively. The adjusted specificity was 16% lower for regions where infectious lung disease is endemic than for those where it is not-61% (95% CI, 49-72%) versus 77% (95% CI, 73-80%). The specificity was also lower when the analysis was limited to rigorously conducted and well-controlled studies. The conclusion is that the data do not support the use of FDG-PET to diagnose lung cancer in areas where infectious lung disease is endemic unless an institution achieves test performance accuracy similar to that found in areas where it is not^(12)^. Because Mosmann et al.^(11)^ did not include these data in their review, is important to highlight that fact.
